# Identification of stress-induced epigenetic methylation onto dopamine D2 gene and neurological and behavioral consequences

**DOI:** 10.36922/gpd.1966

**Published:** 2024-03-29

**Authors:** Kenneth Blum, Abdalla Bowirrat, David Baron, Igor Elman, Milan T. Makale, Jean Lud Cadet, Panayotis K. Thanos, Colin Hanna, Rania Ahmed, Marjorie C. Gondre-Lewis, Catherine A. Dennen, Eric R. Braverman, Diwanshu Soni, Paul Carney, Jag Khalsa, Edward J. Modestino, Debmalya Barh, Debasis Bagchi, Rajendra D. Badgaiyan, Thomas McLaughlin, Rene Cortese, Mauro Ceccanti, Kevin T. Murphy, Ashim Gupta, Miles T. Makale, Keerthy Sunder, Mark S. Gold

**Affiliations:** 1 Department of Molecular Biology, Adelson School of Medicine, Ariel University, Ariel, Israel; 2 Division of Addiction Research & Education, Center for Sports, Exercise & Mental Health, Western University of the Health Sciences, Pomona, CA, United States of America; 3 Institute of Psychology, ELTE Eötvös Loránd University, Budapest, Hungary; 4 Department of Psychiatry, University of Vermont, Burlington, VT 05405, United States of America; 5 Department of Psychiatry, Wright University Boonshoft School of Medicine, Dayton, OH, United States of America; 6 Division of Nutrigenomics, The Kenneth Blum Behavioral Neurogenetic Institute, Austin, TX United States of America; 7 Centre for Genomics and Applied Gene Technology, Institute of Integrative Omics and Applied Biotechnology, Nonakuri, Purba Medinipur, West Bengal, India; 8 Department of Nutrigenomic Research, Victory Nutrition International, Inc., Bonita Springs, FL, United States of America; 9 Division of Personalized Neuromodulation Research, Sunder Foundation, Palm Springs, CA, United States of America; 10 Cambridge Health Alliance, Harvard Medical School, Cambridge, MA, United States of America; 11 Department of Radiation Medicine and Applied Sciences, UC San Diego, 3855 Health Sciences Drive, La Jolla, CA 92093-0819, United States of America; 12 Molecular Neuropsychiatry Research Branch, National Institute on Drug Abuse, National Institutes of Health, Bethesda, MD., United States of America; 13 Behavioral Neuropharmacology and Neuroimaging Laboratory on Addictions, Clinical Research Institute on Addictions, Department of Pharmacology and Toxicology, Jacobs School of Medicine and Biosciences, State University of New York at Buffalo, Buffalo, NY, United States of America; Department of Psychology, State University of New York at Buffalo, Buffalo, NY., United States of America; 14 Department of Anatomy, Howard University College of Medicine, and Developmental Neuropsychopharmacology Laboratory, Howard University College of Medicine, Washington D.C., United States of America; 15 Department of Family Medicine, Jefferson Health Northeast, Philadelphia, PA, United States of America; 16 Division Pediatric Neurology, University of Missouri, School of Medicine, Columbia, MO., United States of America; 17 Department of Microbiology, Immunology and Tropical Medicine, George Washington University, School of Medicine and Health Sciences, Washington, DC, United States of America; 18 Department of Psychology, Curry College, Milton, MA., United States of America; 19 Departamento de Genética, Ecologia e Evolução, Instituto de Ciências Biológicas, Universidade Federal de Minas Gerais, Belo Horizonte, Brazil; 20 Department of Pharmaceutical Sciences, Texas Southern University College of Pharmacy and Health Sciences, Houston, TX, United States of America; 21 Department of Psychiatry, Case Western Reserve University School of Medicine, Cleveland OH., 44106, USA and Department of Psychiatry, Mt. Sinai School of Medicine, New York, NY, United States of America; 22 Department of Child Health – Child Health Research Institute, & Department of Obstetrics, Gynecology and Women’s Health School of Medicine, University of Missouri, MO, United States of America; 23 Alcohol Addiction Program, Latium Region Referral Center, Sapienza University of Rome, Roma, Italy; 24 Division of Personalized Neuromodulation and Patient Care, PeakLogic, LLC, Del Mar, CA, United States of America; 25 Future Biologics, Lawrenceville, Georgia, 30043, United States of America; 26 Department of Psychology, UC San Diego, 3855 Health Sciences Drive, La Jolla, CA 92093-0819, United States of America; 27 Department of Psychiatry, UC Riverside School of Medicine, Riverside, CA, United States of America; 28 Department of Psychiatry, Washington University School of Medicine, St. Louis, MO, United States of America

**Keywords:** Dopamine D2 receptor, Epigenetic modification, Neurological disorders, Stress

## Abstract

The D2 dopamine receptor (*DRD2*) gene has garnered substantial attention as one of the most extensively studied genes across various neuropsychiatric disorders. Since its initial association with severe alcoholism in 1990, particularly through the identification of the *DRD2 Taq A1* allele, numerous international investigations have been conducted to elucidate its role in different conditions. As of February 22, 2024, there are 5485 articles focusing on the *DRD2* gene listed in PUBMED. There have been 120 meta-analyses with mixed results. In our opinion, the primary cause of negative reports regarding the association of various *DRD2* gene polymorphisms is the inadequate screening of controls, not adequately eliminating many hidden reward deficiency syndrome behaviors. Moreover, pleiotropic effects of *DRD2* variants have been identified in neuropsychologic, neurophysiologic, stress response, social stress defeat, maternal deprivation, and gambling disorder, with epigenetic DNA methylation and histone post-translational negative methylation identified as discussed in this article. There are 70 articles listed in PUBMED for DNA methylation and 20 articles listed for histone methylation as of October 19, 2022. For this commentary, we did not denote DNA and/or histone methylation; instead, we provided a brief summary based on behavioral effects. Based on the fact that Blum and Noble characterized the *DRD2 Taq A1* allele as a generalized reward gene and not necessarily specific alcoholism, it now behooves the field to find ways to either use effector moieties to edit the neuroepigenetic insults or possibly harness the idea of potentially removing negative mRNA-reduced expression by inducing “dopamine homeostasis.”

## 1. Introduction

In 2013, Blum’s research team initiated an inquiry into the motivations behind alcohol consumption and high rates of drug-seeking behaviors, particularly in response to stressful situations.^1–4^ Their goal was to raise awareness and understanding of the underlying mechanisms driving such behaviors. Moreover, they posed additional questions regarding the millions of individuals who seek out and engage in high-risk novelty situations, pondering the consequences of pleasure-seeking behaviors. They suggested that the answers to these questions may lie within the intricate workings of our brains and perhaps within our genetic makeup.^5,6^

America is currently facing the worst opioid epidemic in history. In 2021, the centers for disease control and prevention (CDC) reported 106,699 overdose deaths in the US, with 80,441 of those deaths related to opioids. In addition, provisional data from the CDC indicated that opioid deaths in 2022 would rise to approximately 82,998.^7,8^

Blum *et al*. established the concept of reward deficiency syndrome (RDS) in 1995 as a potential predictor of addictive and impulsive behaviors.^5,9–11^ This theory has since been supported by numerous studies, indicating that behavioral, cognitive, and emotional disturbances observed in psychiatric disorders, including RDS, are linked to functional deficits in neurological pathways.^12–17^ For instance, the D2 dopamine receptor (*DRD2*) *Taq A1* allele has been consistently associated with numerous behavioral phenotypes, including aggression,^18^ alcoholism, addictive behaviors,^19^ and neuropsychiatric disorders. Both the *DRD1* and *DRD2* genes are linked to reward pathways and mechanisms.^20^ The culmination of biochemical processes in mesolimbic regions leads to rewarding phenomena, particularly in the nucleus accumbens (NAc), where increased dopamine levels in synaptic spaces interact with *DRD1*, *DRD2*, and other receptor subtypes.^21,22^ Positron emission tomography studies have provided further insights, demonstrating lower *DRD2* availability in individuals with dependence on cannabis, psychostimulants, opioids, or alcohol, as well as those who are obese, compared to control subjects.^23–28^

Furthermore, studies have revealed an association between the A1 and B1 minor alleles of the *DRD2* gene and cocaine use disorder (CUD).^26^ These findings suggest that genetic variations in the *DRD2* gene, located on chromosome 11 at the q22-q23 region, contribute to an increased susceptibility to psychostimulant use disorder (PUD). Interestingly, these observations align with early research by Gold’s group, which hinted at the potential therapeutic efficacy of bromocriptine, a D2 agonist, in combating cocaine abuse and dependence.^29^ Unlike the *DRD2 Taq A1* allele, which is associated with decreased D2 receptor levels, the main variant, *DRD2 Taq A2* allele, is characterized by normal D2 receptor levels, possibly offering protection against psychostimulant misuse and abuse.^30^

Historically, Dackis and Gold were among the first to propose the use of the potent D2 agonist bromocriptine as an epigenetic therapy for severe cocaine dependence.^29^ However, clinical trials revealed that bromocriptine led to the down-regulation of DRD2 receptors and did not prove to be effective for this purpose, resulting in its limited clinical utilization.^31^

This commentary has uncovered a plethora of studies delineating epigenetic alterations occurring within the *DRD2* gene across both substance-related and non-substance-related RDS behaviors.

## 2. Epigenetics and addictive behaviors

Epigenetics refers to the molecular modifications imposed on chromatin within the nucleus of a cell, playing a crucial role in regulating various DNA-related processes, including chromatin organization, DNA repair, RNA transcription, and splicing, among other essential cellular functions.^32^ Substance use disorder (SUD) serves as a prime example of how environmental factors can influence gene expression.^33^ In this regard, a person’s experiences, particularly volitional repetitive drug use, can modify the epigenome in the brain in a way that is specific to certain brain regions and cell types.^34^ It is hypothesized that dysregulation and modification of DNA-related processes induced by drugs can lead to epigenetic alterations, which may contribute to aberrant cellular functions that facilitate the pathogenesis of psychoactive substance dependence. Nestler’s group has previously proposed that gaining insights into epigenetic processes holds therapeutic promise.^35^ Targeting significant drug-induced epigenetic alterations within the brain could potentially disrupt the cycle of drug dependence, thereby preventing individuals from succumbing to a relentless cycle of addiction.^35^

Comprehending the intricacies of the neuroepigenetic landscape necessitates acknowledging the array of epigenetic modifications.^35^ Among these, a significant epigenetic change occurring in adverse environments involves histone post-translational modifications (PTMs), such as methylation and even dopaminylation.^36,37^ Essentially, chromatin consists of DNA that is intricately wound around histone protein octamers and forms nucleosomes, which subsequently allows for compact packaging within the cell nucleus. This structural arrangement serves as an adaptable scaffold that responds to external stimuli. Notably, histones are rich in arginine and lysine residues, contributing to their highly basic nature. PTMs of these residues and others on histone N-terminal tails, extending from the nucleosome core, modulate the physical properties and charge distribution of chromatin, thereby regulating DNA-associated processes.

Histone subunits undergo a multitude of PTMs, including but not limited to acetylation, methylation, phosphorylation, adenosine diphosphate ribosylation, ubiquitylation, and sumoylation, with an expanding array of newly identified modifications.^38,39^ These modifications occur on over 50 distinct sites across histone proteins.^38,39^

According to Allis *et al*., “histone PTMs are reversible; they are dynamically deposited by “writer” enzymes, recognized by “reader” proteins which mediate the cellular response, and removed by “eraser’ enzymes.”^36^ Studies have demonstrated that the expression and activity of numerous writer, eraser, and reader proteins are dysregulated in both addicted individuals and animal models of addiction.^40–42^ This dysregulation has spurred interest in the development of novel epigenetic therapies for addiction. The restoration of normal function to these proteins, either through small molecule interventions or functional food complexes that help rebalance neurotransmitter levels, such as dopamine, represents a promising avenue for anti-addiction epigenetic treatments.^43–52^

## 3. Epigenetic biomarkers

### 3.1. DNA methylation

A biomarker is a quantitative, measurable indicator of a biological molecule, state, or condition.^53^ DNA methylation is the most researched epigenetic biomarker due to its chemical stability, role in mammalian development and disease, and its significant role in modulating gene expression across a wide array of biological processes.^54,55^ Although DNA methylation induces physical changes to gene structure, it is reversible in nature. Its primary function is to impede DNA transcription, thereby suppressing the expression of specific genes. DNA methylation tests have become more affordable and accessible and require only a small amount of DNA, which can be obtained from body fluids, cells, or tissues.^19^ In addition, DNA can be isolated from bacteria, viruses, plants, or mammals. DNA methylation is a biochemical process characterized by the addition of a methyl group to DNA molecules. A common occurrence is the addition of a methyl group to the 5-carbon position of a cytosine ring, forming 5-methylcytosine (5-mC).^56^ DNA methylation assays are techniques employed to quantify the levels of 5-mC within DNA samples. The enzyme DNA methyltransferase (DNMT) plays a pivotal role in catalyzing DNA methylation, particularly at CpG dinucleotide sites.^57^ Notably, DNMT-1 is primarily responsible for DNA replication in the mitotic cells of the brain, which exhibit the highest levels of DNA methylation in the body.^58^ DNMT-3A and DNMT-3B, on the other hand, regulate methylation patterns during early developmental stages.^59^

NA methylation plays a critical role in early brain development and the specification of regions through gene expression.^57^ It also significantly influences mutational events associated with various cancers, increasing the risk of gene mutations and the inactivation of specific tumor-suppressor genes.^60–64^ For instance, exposure to environmental carcinogens, such as pollution, can induce mutations in genes responsible for DNA methylation, leading to altered cellular states such as proliferation or differentiation and ultimately resulting in cancer.^64^

Moreover, DNA methylation is involved in genomic imprinting, a process where genes are silenced or inactivated through DNA methylation. Imprinting occurs when one allele from either the father or mother is silenced, resulting in an imprinted gene.^65^ These parent-of-origin effects can be inherited by gametes and passed down to offspring, giving rise to various diseases such as Prader-Willi syndrome and Angelman syndrome.^66,67^ This mode of inheritance also aids in understanding the role of DNA methylation in psychiatric disorders such as major depressive disorder, bipolar disorder, schizophrenia, autism, and related conditions. This finding is significant as it provides crucial insights into how abnormalities in this mechanism contribute to the pathophysiology of diverse disorders, and it suggests the potential of DNA methylation as a therapeutic target.^67^

### 3.2. DNA demethylation

DNA demethylation is a process that occurs alongside DNA methylation but is not as widely understood.^68^ It serves as a biomarker for DNA damage, involving the removal of a methyl group from DNA, which can occur actively or passively in both dividing and non-dividing cells. Passive demethylation entails the loss of 5mC during DNA replication, while active demethylation involves the alteration or removal of a methyl group from 5 mC. Notably, 5 mC, a methylated form of cytosine, is commonly utilized as a point of interest for gene mutations and as an epigenetic marker due to its regulatory role in gene transcription.^55,69^

Furthermore, a derivative of 5mC known as 5-hydroxymethylcytosine (5hmC) is abundantly present in various organ tissues, particularly in the brain. DNA demethylation acts as a marker for DNA damage and facilitates repair processes by identifying potentially mutated sites.^70^ Primordial germ cells of an embryo and developing zygotes are the primary sites where demethylation occurs, emphasizing its key role in differentiation mechanisms.^71^

DNA demethylation is mediated by enzymes from the ten-eleven translocation (TET) family.^72,73^ The TET enzymes function as tumor suppressors in various malignancies, and their loss or dysfunction is closely associated with rapidly mutating cancers.^74^ In addition, thymine DNA glycosylase plays a crucial role in DNA demethylation and normal development by initiating base excision repair, which is essential for repairing damaged DNA throughout the cell cycle.^75^

In summary, DNA demethylation serves as a mechanism for epigenetic reprogramming of genes, influenced by environmental risk factors such as injuries and substance use.

### 3.3. Histone modification

Histones, a class of proteins, facilitate the packaging of DNA into structural units known as nucleosomes. This packaging provides structural support and ensures that DNA fits appropriately within the nucleus of the cell. The modification of histones exemplifies epigenetic regulation, as it influences transcription and alters phenotypes in response to environmental stimuli and stressors. Histone modifications achieve this process by tightly compacting DNA, hindering its accessibility to the cellular machinery.^76^ Conversely, histone relaxation facilitates increased access of proteins to DNA, thereby enhancing its susceptibility to analysis by the cell. Histone alterations have also been found to affect DNA repair and replication, in addition to cell state modifications.^77^ They are also utilized to synthesize macromolecules like lipids and carbohydrates, as well as to regulate cell metabolism and energy outputs.^78^ It is crucial to note that histone alterations frequently have an indirect effect by activating downstream signaling cascades through proteins.

### 3.4. Histone acetylation

Histone acetylation represents a reversible epigenetic mechanism often associated with enhanced gene transcription and implicated in processes underlying memory formation and drug addiction. It serves as a conduit for environmental cues, particularly those from drugs of abuse, to elicit targeted modifications in gene expression. The enzymatic regulation of acetylation involves histone deacetylases (HDACs) and histone acetyltransferases (HATs).^79^ During histone acetylation, a negatively charged acetyl group is affixed to lysine residues on histone proteins,^80^ resulting in a relaxed chromatin structure conducive to transcription factor binding and heightened gene expression.

Among histones, H3 and H4 are highly conserved and have garnered significant attention due to their pivotal roles in chromatin organization.^81^ Histone acetylation often occurs concomitantly with other chromatin modifications. Similar to DNA methylation and demethylation, histone acetylation is anticipated to function as a biomarker for various diseases. A reduction in histone acetylation has been associated with neurodevelopment disorders, neural degeneration, plasticity, and memory impairment. The CLOCK protein exemplifies HAT and stands as a prominent transcription factor integral to circadian rhythms and cellular homeostasis. Disruptions in this HAT cause a number of changes associated with sleep deprivation and bipolar disorder manic-like behaviors.^82^ A thorough investigation into histones will be essential for the development of numerous therapeutic strategies for psychiatric disorders, neurodegenerative diseases, and tumor cells.

### 3.5. Chromatin remodeling

Changes to histone proteins can influence the structure of chromatin, encompassing modifications such as acetylation, deacetylation, methylation, and demethylation.^83^ These modifications ultimately dictate whether transcription is activated or repressed. Chromatin that is tightly condensed and transcriptionally inactive is termed heterochromatin, where genes are typically silenced or inactivated. Conversely, euchromatin refers to loosely condensed chromatin that is more accessible for transcription. In euchromatin, DNA is readily accessible for binding by transcription factors and other DNA-binding proteins, facilitating the regulation of gene expression.^84^ The process of transitioning chromatin from a condensed to a more accessible state is referred to as chromatin remodeling, as discussed in earlier sections detailing histone modifications.

Chromatin plays a pivotal role in a multitude of cellular functions, encompassing DNA repair, DNA replication, chromosome segregation, and signal transduction, among others. Chromatin remodelers typically consist of multiprotein complexes and enzymes that harness adenosine triphosphate hydrolysis to modify histones and reconfigure nucleosomes. An example of chromatin remodeling is evident in the development of an embryonic heart. Mutations or defects resulting from these remodelers can lead to a variety of cardiovascular disorders in adults.^85^

### 3.6. Non-coding RNA (ncRNA) expression

ncRNA refers to RNA molecules transcribed from DNA but not translated into proteins. There are four major types of ncRNA involved in regulating gene expression: MicroRNAs (miRNA), long ncRNAs (lncRNA), Piwi-interacting RNAs, and short interfering RNAs, all of which have been extensively investigated.^86^ These regulatory mechanisms play pivotal roles in managing transcription and translation, influencing chromatin remodeling, and are integral to physiological processes as well as disease states.^87^ MiRNAs function by binding to target RNAs, thereby suppressing gene expression and inhibiting translation. They facilitate the transfer of genetic information within cells, between different cells and tissues, and even across body fluids such as breast milk, sweat, urine, and blood. Due to their involvement in cardiovascular disorders and tumor suppression, miRNAs are considered excellent diagnostic markers.^60,64^ In contrast, lncRNAs are more celltype specific and are expressed at lower levels compared to miRNAs. They primarily regulate transcription, which may occur within or outside the nucleus. In addition, lncRNAs exhibit diverse functions, including regulation of mRNA within the cytoplasm of a cell.^86^

## 4. The *DRD2* gene as a therapeutic target for RDS

In a blinded study, Blum *et al*.,^88^ demonstrated the initial allelic association of the *DRD2* gene with alcoholism. The study employed 70 brain samples collected from both non-alcoholics and individuals diagnosed with alcoholism. DNA samples underwent digestion with restriction endonucleases and were probed with a clone containing the complete 3’ coding exon, the polyadenylation signal, and approximately 16.4 kilobases (kb) of non-coding 3’ sequence of the human *DRD2* gene (lambda hD2G1). The findings revealed that the presence of the A1 allele of the *DRD2* gene accurately classified 77% of the individuals with alcoholism, while its absence correctly classified 72% of the nonalcoholics. This polymorphic pattern of the *DRD2* gene suggested the presence of a susceptibility gene for at least one form of alcoholism on the q22-q23 region of chromosome 11.

Using similar brain tissue, Noble *et al*.^23^ conducted a study investigating the allelic association of the human *DRD2* gene with the binding characteristics of the *DRD2* receptor in 66 brains obtained from both non-alcoholic and alcoholic subjects. In this blinded experiment, DNA extracted from the cerebral cortex underwent treatment with the restriction endonuclease Taq and was probed with a 1.5-kb digest of a clone (XhD2G1) of the human *DRD2* gene. The binding characteristics, including the binding affinity (K_d_) and the number of binding sites (B_max_), of DRD2 were determined in the caudate nuclei of these brains using tritiated spiperone as the ligand. The results revealed that compared to nonalcoholic subjects, the adjusted K_d_ was significantly lower in alcoholic subjects, indicating a higher binding affinity of DRD2 in the latter group. In addition, B_max_ was found to be lower in subjects carrying the A1 allele, which exhibited a strong association with alcoholism, whereas no significant changes in B_max_ were observed in subjects carrying the A2 allele. Moreover, a progressively reduced B_max_ was found in subjects with A2/A2, A1/A2, and A1/A1 alleles, with subjects carrying A2/A2 alleles exhibiting the highest mean values and those with A1/A1 alleles exhibiting the lowest. The observed polymorphic pattern of the *DRD2* gene and its variation in receptor expression strongly suggests the involvement of the dopaminergic system in predisposing individuals to at least one subtype of severe alcoholism. Subsequent studies have corroborated these findings,^89–104^ and as of February 22, 2024, a PubMed search using the term “Dopamine D2 Receptor Gene” yielded a total of 5485 listings.

While we are cognizant of epigenetic insults affecting at least seven major neurotransmitter pathways, including cannabinoidergic, cholinergic, serotonergic, opioidergic, gluconergic, GABAergic, and glutaminergic, our focus lies on the dopaminergic system, specifically the *DRD2* gene. The primary review, which is based on PUBMED listings using the search term “Methylation and the Dopamine D2 Receptor Gene,” yielded 378 listings as of February 22, 2024.

## 5. Epigenetic modifications and *DRD2* gene

While we recognize that many reward genes and associated loci have been shown to undergo epigenetic modifications after the consumption of drugs of abuse (i.e., alcohol), the purpose of this commentary is to present a narrative that focuses on identifying epigenetic modifications, specifically in the *DRD2* gene. In this commentary, we chose not to denote DNA and/or histone methylation, but instead, we provide a summary based on behavioral effects.

A recent study by Bohnsack *et al*. provides an example of targeting and editing epigenetic histone modifications to attenuate unwanted behaviors.^104^ Adolescent binge drinking is widely recognized to induce epigenetic modifications at the enhancer region of the activity-regulated cytoskeleton-associated protein (ARC) immediate-early gene, specifically known as the synaptic activity response element (SARE).^104^ These epigenetic modifications lead to a decrease in *ARC* expression in the amygdala, a phenomenon observed in both rodent models and human studies.^104^

In an experiment conducted by Pandey’s group, it was demonstrated that the use of dCas9-P300 resulted in increased histone acetylation at the *ARC* SARE. This intervention effectively normalized deficits in *ARC* expression and consequently led to a reduction in adult anxiety and excessive alcohol consumption in a rat model that examined alcohol exposure during adolescence.^104^ Interestingly, dCas9-Kruppel-associated box (KRAB), in contrast, was found to promote repressive histone methylation at the *ARC* SARE, resulting in decreased *ARC* expression, the manifestation of anxiety-like behaviors, and increased alcohol consumption in the control group.

### 5.1. SUD

A summary of studies focusing on the epigenetics SUD is presented in Table 1. Detailed descriptions of SUD are provided in the following subsections.

#### 5.1.1. Cannabis

In a study conducted by Oyaci *et al*.,^105^ DNA methylation levels at the *DRD2* gene were compared among patients based on clinical parameters and *DRD2* genotype distribution. Their findings revealed significant differences in methylation status between groups, particularly concerning the presence of a family history of CUD or synthetic cannabinoid use disorder (SCUD). One notable limitation of the study is the utilization of inaccurate controls (the authors did not screen the controls for other potentially addictive behaviors such as gambling and overeating). It is known that without such screening, the presence of the *DRD2 A1* allele, for example, could be as high as 40%. This flaw could have prevented the investigators from establishing direct evidence for high DNA methylation with both CUD and SCUD. In a separate work conducted by Gerra *et al*.,^106^ a genetic and sociodemographic analysis involving both CUD patients and control groups was performed to explore DNA methylation in the *DRD2-ANKKI* gene region. They identified significant hypermethylation at exon 8 of the *DRD2* gene. Interestingly, the study also uncovered a correlation between higher education levels and a reduced risk of CUD. The authors astutely proposed that their findings of differentially methylated regions (DMRs) at exon 8 of the *DRD2* gene could serve as potential biomarkers or targets for the development of pharmacological therapeutic agents.

In a study by DiNieri *et al*.,^107^ utilizing an animal model of prenatal exposure to delta-9-tetrahydrocannabinol (THC), it was observed that prenatal cannabis exposure led to a reduction in *DRD2* messenger RNA expression in the NAc, a crucial brain reward region. Furthermore, Hurd’s group^107^ identified histone methylation differences in the offspring of pregnant rats exposed to THC. Chromatin immunoprecipitation of the adult NAc revealed an increase in the 2meH3K9 repressive mark and a decrease in 3meH3K4 and RNA polymerase II at the *DRD2* gene locus in the THC-exposed offspring. This diminished *DRD2* expression correlates with reduced *DRD2* binding sites and heightened sensitivity to opiate reward in adulthood. Moreover, the study suggested that maternal cannabis use alters the epigenetic mechanisms governing histone lysine methylation, thereby influencing the developmental regulation of mesolimbic *DRD2* in offspring. This subsequent reduction in *DRD2* may increase the susceptibility of the offspring to addiction in the future. Of interest, earlier work from Blum’s laboratory^108^ demonstrated significant alteration of the vas deferens from prenatal exposure to THC in that there was an increased sensitivity to enkephalins, in agreement with the findings of DiNieri *et al*.^107^

#### 5.1.2. Tobacco

As highlighted by Liu *et al*.,^109^ previous research has underscored the importance of the *NCAM1-TTC12-ANKK1-DRD2* gene cluster in addiction susceptibility among individuals of European and African descent. Building on this foundation, Liu and colleagues conducted next-generation bisulfite sequencing to uncover smoking-associated DMRs. Through haplotype-based association analysis, they identified a significant association between cigarettes per day and the C-T-A-G haplotype in *DRD2*, formed by rs4245148, rs4581480, rs4648317, and rs11214613. Furthermore, the study identified four significant smoking-associated DMRs, with three residing in the *DRD2/ANKK1* region. These findings elucidate a notable association of variants and haplotypes within the *ANKK1/DRD2* region with nicotine dependence among Chinese male smokers. Importantly, the results also underscore the pivotal role of DNA methylation in facilitating such associations.

#### 5.1.3. Alcohol

Strong correlations have been observed between DRD2 binding potential and neural responses to rewarding stimuli and substance use.^89–104^ Consequently, disruptions in DRD2 function constitute a critical component of theoretical models elucidating the pathophysiology of substance dependence.^110^ Moreover, as proposed by Pandey *et al*.,^111^ epigenetic modifications may serve as fundamental molecular mechanisms influencing how alcohol exposure impacts the brain.

This idea has been investigated by Bidwell *et al*., who demonstrated that, after controlling for age, *DRD2* promoter DNA methylation was positively associated with responses to alcohol cues in the left caudate, right caudate, left putamen, right putamen, and right Nac.^77,112^ This finding indicated that robust striatal activation, in response to reward cues, was linked with DNA methylation at the *DRD2* gene. In addition, *DRD2* methylation was linked to alcohol use disorder (AUD) severity. Specifically, *DRD2* methylation was linked to scores on the AUDs Identification Test, Impaired Control Scale, and Alcohol Dependence Scale.

Chronic consumption of alcohol and various other illicit drugs has been associated with adverse consequences, including functional connectivity deficits within neural networks linked to executive control.^113^ These deficits may indirectly contribute to the development of aberrant alcohol-seeking behavior.^113^ Hagerty *et al*.^114^ found that, specifically, average DNA methylation at the *DRD2* gene was negatively correlated with left and right executive control network connectivity, but no correlation was found with the other networks that were tested. In addition, *DRD2* DNA methylation was found to be linked with the severity of alcohol problems. These findings bolster a theoretical framework linking the neurobiological markers of alcohol consumption among polysubstance users to epigenetic influences.

Individuals with AUD tend to have dopaminergic alteration, and those with a family history of AUD may experience influences on brain development.^115^ Hill and Sharma discovered a significant positive correlation between familial high-risk status and DNA methylation at the *DRD2* gene.^116^ In fact, significant differences in the volume of the left inferior temporal, left fusiform, and left insula regions were observed between high-risk and low-risk familial risk groups. Previously, these regions have been associated with social cognition. In addition, the DNA methylation of the *DRD2* gene was inversely correlated with the volumes of grey matter in these regions.

Along similar lines of thinking, Hillemacher *et al*.,^117^ concerned about the role of epigenetics on dopaminergic neurotransmission and its influence on alcohol dependence, found a significant increase of DNA methylation at the *DRD2* gene during alcohol withdrawal/early abstinence. Furthermore, they discovered a significant association between craving, as measured by the obsessive-compulsive drinking scale, and the DNA methylation of the *DRD2* gene. Their findings regarding this association with alcohol craving underscore the pivotal role of *DRD2* gene DNA methylation in the neurobiology of addictive behavior. It is worth noting that the inability to demonstrate significant alterations in DNA methylation between controls and patients may stem from an inadequate screening of the control group.^118^

Moreover, as previously pointed out by several researchers,^89–104^ reduced ligand binding of DRD2 has consistently been observed in the striatum of individuals with AUD and other RDS behaviors. The reduced DRD2 binding has been suggested to indicate diminished DRD2 density, which, in turn, has been proposed to trigger cravings and increase the likelihood of relapse. Accordingly, Feltmann *et al*.,^119^ surprisingly, unlike others, did not find DNA methylation differences in the investigated regions of the *DRD2* gene. However, in Wistar rats, chronic alcohol drinking significantly decreased mRNA levels of the long isoform of the *DRD2* gene in the NAc. In addition, alcohol drinking also decreased the striatal density of DRD2-DRD2 homoreceptor complexes, increased the density of A2AR-DRD2 heteroreceptor complexes in the NAc shell and the dorsal striatum, and decreased the density of sigma1R- DRD2 heteroreceptor complexes in the dorsal striatum. Interestingly, the chronic alcohol consumption in these rats appears to fit well with earlier findings from Blum’s laboratory involving Golden Syrian hamsters.^120^ In particular, compared to the control hamsters who only drank water, the experimental hamsters who freely drank ethanol after a year had a noticeably lower concentration of a leucine-enkephalin-like immunoreactive substance in their basal ganglia.^120^

This discovery suggests that ethanol’s effects involve the synthesis of endogenous peptidyl opiates, which appears akin to the findings of long-term drinking, as presented in the Feltmann *et al*. study. Thus, although we cannot rule out the possibility of DNA methylation differences occurring in other regions and/or other epigenetic modifications not studied in the Feltmann *et al*.^119^ study, it begs the question as to the possibility that mRNA expression suppression by alcohol drinking also reduces the synthesis of DRD2. Feltmann *et al*.^119^ also provided support for the hypothesis that AUD was associated with a hypodopaminergic system and proposed the A2AR-DRD2 heteroreceptor complex as a promising novel target for treatment.

#### 5.1.4. Psychostimulant abuse

Recently, Blum *et al*.^30^ proposed that mild D2 receptor stimulation achieved by certain therapeutic modalities can cause dopamine release, which, in turn, can change D2-directed mRNA and improve DRD2 function in humans. This increase in DRD2 activity is thought to reduce craving behaviors, particularly in high-risk, genetically compromised populations. Accordingly, Cadet’s group has conducted eloquent experiments comparing methamphetamine (METH) and modafinil in terms of epigenetic insults.^121^

González *et al*.,^121^ demonstrated that repeated administration of either METH or modafinil to mice resulted in cognitive effects. In the medial prefrontal cortex, there were observed inductions in histone acetylation and DNA methylation profiles. Mice subjected to repeated METH exposure, but not modafinil, exhibited impaired cognitive memory, as revealed by the novel object recognition test, with a notable decrease in memory recognition. In addition, METH-treated mice demonstrated (i) decreased levels of histone H3 and H4 acetylation and increased levels of 5-mC and (ii) reduced histone H3 acetylation enrichment at promoters of the *DRD2* gene. These findings suggest that epigenetic dysregulation, particularly at the *DRD2* gene, is associated with the long-term cognitive decline effects of METH and its adverse impacts on medial prefrontal cortex function. Further studies are warranted to elucidate the specific mechanisms of epigenetic regulation affected by psychostimulant abuse.

Changes in histone methylation and acetylation on DNA within the NAc and lysine residues have been observed with repeated cocaine administration. Nestler’s group investigated histone arginine (R) methylation in models related to reward processing. Specifically, Damez-Werno *et al*.^122^ found that in both animal and human self-administration experiments, the histone mark protein-R- methyltransferase-6 (PRMT6) and asymmetric demethylation of R2 on histone H3 (H3R2me2a) were reduced in the rodent and cocaine-dependent human NAc. In fact, while PRMT6 overexpression in medium spiny neurons (MSNs) expressing DRD1 (D1-MSNs) is protective against cocaine-seeking behaviors, PRMT6 overexpression in D2-MSNs in all NAc neurons has been associated with increased cocaine-seeking behaviors. Along these lines, Blum *et al*. hypothesized that dopaminylation (H3R2me2a binding) occurs in PUD, and the binding inhibitor Srcin1, like the major *DRD2 A2* allelic polymorphism, protects against psychostimulant-seeking behavior by normalizing NAc dopamine expression. Moreover, numerous studies have verified the association between the *DRD2 Taq A1* allele (30 – 40 lower DRD2 numbers) and severe cocaine dependence. According to Lepack *et al*.,^123^ acute cocaine increases dopamine in NAc synapses and causes histone H3 glutamine 5 dopaminylation and subsequent inhibition of *DRD2* expression. With prolonged cocaine use, the inhibition of *DRD2* expression increases and accompanies cocaine withdrawal. Furthermore, they have reported that during cocaine withdrawal, the Src kinase signaling inhibitor 1 (Srcin1 or p140CAP) decreased H3R2me2a binding. Thus, this inhibited dopaminylation induced a “homeostatic brake.” Blum’s group^37^ suggested that the reduction in Src signaling in NAc D2-MSNs, like the *DRD2 Taq A2* allele, a well-known genetic mechanism protective against SUD, normalizes the NAc dopamine expression and decreases cocaine cravings and cocaine-seeking behaviors. Therefore, Srcin1 could be an important target for therapeutic interventions.

Another important facet related to METH-induced psychosis may involve both genetic DNA antecedents and epigenetic post-translational changes in mRNA expression, for example, on the *DRD2* gene. Nohesara *et al*.^124^ found that METH induces a decrease in DNA methylation of a number of dopamine-related genes (i.e., *DRD3*, *DRD4*, and *COMT*) in patients with METH psychosis but not in non-METH psychosis patients. The suggestion here is that, in general, METH dependency is linked with a reduction in DNA methylation and an open chromatin conformation, leading to increased expression of several important genes involved in the pathogenesis of psychotic disorders.^121^ Nohesara *et al*.^124^ suggested that these epigenetic modifications may serve as valuable diagnostic biomarkers for diagnosing psychosis in METH abusers. In addition, they support the use of a methyl-rich diet for the suppression or prevention of psychosis in these patients, thus encouraging further association and interventional studies involving larger populations.

#### 5.1.5. Opioid abuse

Opioid use disorder (OUD) and other reward-dysregulated disorders have a high degree of heritability. Zhang *et al*.^125^ demonstrated that various methylation quantitative trait loci (mQTLs) in the *DRD1* and *DRD2* genes were identified in both the healthy control and heroin use disorder groups. Specifically, rs4867798-CpG_174872884 and rs5326-CpG_174872884 in the *DRD1* gene were the unique single-nucleotide polymorphism-CpG pairs observed in patients suffering from heroin use disorder. This groundbreaking research suggests that certain dopaminergic mQTLs may be linked with characteristics of OUD by implicating DNA methylation and gene expression. However, it underscores the necessity for improved screening of controls to reassess the findings regarding DRD2.^120^

#### 5.1.6. Eating disorders

Similar to SUD, anorexia nervosa is a multifaceted and highly heritable disease.^126–128^ Rask-Andersen *et al*.^128^ reviewed the relevant literature and discovered that 175 association studies had been conducted on an anorexia nervosa cohort, examining 128 different polymorphisms related to 43 genes. The strongest correlations indicate that certain dopaminergic genes play a key role in regulating body mass index.^129^ Moreover, findings by Frieling *et al*.^130^ revealed an increase in the expression of dopamine transporter (*DAT*) mRNA and a decrease in *DRD2* expression. Frieling *et al*. suggested that the upregulation of the *DAT* gene was accompanied by a hypermethylation of the gene’s promoter in the anorexia nervosa and bulimia nervosa group, while significant hypermethylation of the *DRD2* promoter was only present in the anorexia nervosa group. No discrepancies in methylation or expression were detected in the various other dopamine receptors that were examined. In addition, Groleau *et al*.^131^ found that women diagnosed with bulimia spectrum disorder, particularly those with borderline personality disorder, demonstrated significant elevations in *DRD2* DNA methylation levels than women with no eating disorders. Moreover, women with a history of bulimia spectrum disorder and childhood sexual abuse showed increased *DRD2* DNA methylation compared to the group with no eating disorder history. These results suggest that individuals with bulimia spectrum disorder experience an increase in DNA methylation of the *DRD2* gene promoter, which may serve as a stronger marker of comorbid psychopathology rather than being a board correlate of eating disorders in general.

#### 5.1.7. Gambling

According to several studies, dopaminergic circuits may play a role in the pathophysiology of problematic gambling behavior.^132–134^ Hillemacher *et al*.^134^ reported that DNA methylation patterns in the *DRD2*-gene were modified with respect to abstinence over a 12-month or a 30-month period. In addition, they observed increased levels of DNA methylation in individuals who were non-abstinent and those who did not seek treatment. These findings suggest that altered *DRD2* expression, resulting from variations in DNA methylation, holds pathophysiological significance in lifetime pathologic gambling behavior.

#### 5.1.8. Personality disorders

Utilizing chimpanzees, which are known to exhibit five dimensions of personality (agreeableness, dominance, extraversion, openness, and reactivity/undependability), Staes *et al*.^135^ observed that *DRD2* DNA methylation is most strongly linked to extraversion. In addition, they found that varying DNA methylation levels at specific *DRD2* sites were linked to changes in extraversion in nursery-reared, but not mother-reared, individuals. These findings provide further support for the importance of biological mother-related rearing in early life. This work further suggests that early-life experiences can influence long-lasting behavioral effects, theoretically through epigenomic modification. Accordingly, these results contribute to the increasing body of research demonstrating the critical role of the experience-dependent methylome in the development of personality.^134,136–140^

#### 5.1.9. Maternal deprivation (MD)

Gondre-Lewis’s group correctly points out that stress experienced early in life can trigger a complex neurochemical cascade, affecting the emotional and addictive behaviors of adolescents and adults later in life.^141^ Moreover, research indicates that drug-seeking behavior and excessive alcohol drinking are often comorbid with depressive-like symptoms and behaviors.^142^ These behaviors are frequently observed in individuals who faced adversity in their early life and are important features found in animal models of early life stress exposure.^143^ In fact, Guo *et al*.^144^ reported that, in rats, the rats subjected to MD, chronic unpredictable stress (CUS), and a combination of both (MD/CUS) displayed increased levels of *DRD2* promoter DNA methylation compared to normal controls. The authors concluded that early-life MD increased vulnerability to stress-induced depressive-like behavior in adult rats. In line with the findings of Gondre-Lewis’s group,^141–143^ increased methylation of the *DRD2* promoter gene in the ventral tegmental area may raise the risk of depression and alcohol-seeking behavior. Furthermore, Zhu *et al*.^145^ explored the effects of MD on adult rats’ spatial learning and memory, exploratory, and limbic activity, along with their association with the expression of *DAT*, *DRD1*, *DRD2*, and *DRD3* in the NAc. In addition, Zhu *et al*.^145^ investigated the potential involvement of DNA methylation in the regulation of *DRD2* gene expression. Based on their work, only the *DRD2* mRNA level was associated with total distance. However, the expression of DNMT1 and 3 alpha (DNMT3A) and the methylated CpG levels in the promoter region of the *DRD2* gene were not significantly altered in the MD group compared to the control group.^120^ Functional differences may have occurred in other sites at the *DRD2* genes not included in the study, and/or other epigenetic modifications were associated with *DRD2* mRNA expression. Notwithstanding, it is possible that deficient control screening and interpretation may have impacted the association study, and further research with carefully selected controls, not just MD, may alter these results.

#### 5.1.10. Social defeat stress

In terms of social defeat stress, Zhu *et al*. reported that chronic social defeat stress regulates FOSB expression in the NAc, which promotes the cell-type-specific accumulation of ΔFosB in the two MSN subtypes in this region. ΔFosB is selectively induced in D1-MSNs in the NAc of resilient mice and in D2-MSNs of susceptible mice.^146^ In this regard, Hamilton *et al*. reported that FOSB-targeted histone acetylation in D2-MSNs or histone methylation in D1-MSNs promotes a stress-susceptible, depressive-like phenotype, while histone methylation in D2-MSNs or histone acetylation in D1-MSNs enhance resilience to social stress as measured by social interaction behavior and sucrose preference.^147^ Importantly, the study presented the first evidence of targeting histone modifications specifically to genes and cells, simulating real-world transcriptional processes that regulate social defeat stress behavior.

## 6. Effector moieties

The effector moieties are molecules that selectively bind to proteins to modify their biological activities. These molecules, essentially ligands, can modify enzyme activity, genomic activity, cell signaling, and other protein functions.^35^ While identifying epigenetic-induced post-transcriptional methylation or acetylation on chromosome histones is crucial, one of the first and most successful effector moieties in editing transcriptional activator domains was VP64, which is derived from the herpes simplex virus. VP64 recruits RNA polymerase ll directly for gene-targeted transcriptional activation.^148^ It is now understood that by targeting VP64 to the promoter region of a single gene using DNA-binding domains such as ZFP,^149^ TALE,^150,151^ or CRISPR/dCas9,^152^ laboratories can consistently induce transcription of targeted genes *in vitro* and even *in vivo*.^153,154^

Furthermore, to attain bidirectional control over gene expression, the suppression of endogenous genes is typically achieved using KRAB effector moieties. KRAB represents a transcriptional repression domain present in human zinc finger transcription factors. It functions by recruiting heterochromatin-forming complexes that subsequently deposit H3K9me3 repressive marks, resulting in transcriptional suppression.^155^ Similar to VP64, the KRAB domain modifies the epigenome by enlisting the secondary factors rather than utilizing enzymatic activity. In addition, promoter- or enhancer-targeted KRAB has been used in cell culture and in the brain.^156,157^ In addition, research has identified specific transcription factors and epigenetic readers, erasers, and writers associated with the addiction pathogenesis. More recently, a number of effector moieties have been developed for neuroepigenetic editing. These effector moieties include DNA-modifying enzymes such as DNMT3A (responsible for CpG methylation).^158,159^ and TET1 (which catalyzes hydroxymethylation of CpG).^160,161^ They also encompass proteins involved in regulating histone PTMs such as NFκB subunit p65 (which recruits HATs to acetylate core histones).^162^ histone methyltransferase G9a (which catalyzes H3K9me2),^162,163^ p300 HAT (which acetylates all four core histones),^164^ Sin3-interaction domain (which recruits HDACs),^165,166^ lysine-specific demethylase 1 (which demethylates H3K4 and H3K9),^167^ PRDM9 (which methylates H3K4 and H3K36),^168^ and DOT1L (which methylates H3K79).^169,170^ Furthermore, transcription factors such as CREB have also been utilized, thus mimicking known mechanisms of transcriptional regulation.^171^

## 7. Research outlook in epigenetics of SUD

The evidence presented in this commentary strongly indicates the significant role of epigenetic (mis) regulation in SUD. Numerous associations and interventional studies demonstrate that epigenetic mechanisms are responsible for the complex regulation of dopamine homeostasis, particularly concerning the expression of the *DRD2* gene. Epigenetic regulatory mechanisms (i.e., histone modifications, ncRNA, and DNA methylation) interact in an orchestrated fashion to establish phenotypes influenced by genetic background (e.g., the presence of mutations) and environmental factors (e.g., the presence of active compounds from drugs, alcohol, and tobacco). Importantly, these interactions occur in a tissue- and cell-specific manner. Hence, a detailed mapping of epigenetic modifications across various presentations of SUD will enable the understanding of the precise regulatory mechanisms involved in each case and identify potential targets for therapeutic interventions.

The utilization of effector moieties emerges as a promising intervention. However, caution is warranted due to the pleiotropic effects of epigenetic modifiers (e.g., DNMTs, HDACs, and HTAs), and systemic inactivation of these enzymes may lead to undesirable side effects, hindering their clinical translation. Further research is needed to elucidate mechanisms for cell-specific activation or delivery of the effector moieties to mitigate side effects. Moreover, given that prenatal SUD may impact offspring health, mechanistic and interventional studies are imperative to determine and target epigenetic modifications, aiming to mitigate the effects of prenatal SUD on fetal health.

## 8. Summary

SUD often entails persistent behavioral abnormalities observed in susceptible individuals following repeated exposure to psychoactive drugs of abuse. The enduring nature of these behavioral alterations suggests potential long-lasting changes in gene expression within specific brain regions, which may contribute to the addiction phenotype. Advanced research over the past decade has revealed the pivotal involvement of epigenetic mechanisms in orchestrating lasting alterations, whether positive or negative, in gene expression across various tissues, particularly in the brain. This understanding has spurred investigations aimed at elucidating the impact of epigenetic regulatory processes in mediating the enduring effects of psychoactive substances of abuse on the brain, predominantly using animal models of drug addiction. However, more recently, human neuroepigenetic research has been rapidly emerging. Compelling evidence indicates that repeated exposure to drugs of abuse induces alterations within the brain’s reward regions through three primary modes of epigenetic regulation: Histone modifications such as acetylation and methylation, DNA methylation, and ncRNAs. In this commentary, our focus lies on investigating epigenetic modifications to the *DRD2* gene to directly illustrate the role of these epigenetic changes in addiction-related behavioral abnormalities. At present, there is a growing awareness regarding the utilization of various effector moieties to counteract these undesirable negative epigenetic changes, as recently observed by Pandey’s group^104^ in mitigating alcohol-induced anxiety.

Experimentally, effector moieties are providing unprecedented information relevant to not only mechanistic insights but also to demonstrate the importance of removing these negative epigenetic insults, leading to the concomitant attenuation of specific mRNA transcriptional expression, such as the *DRD2* gene. One important unanswered question is whether induction, for example, of dopamine homeostasis, would increase the conversion of post-transcriptional histone methylation to acetylation. Our laboratory has been developing a nutraceutical complex to gently induce pro-dopamine regulation, potentially in both animal models and humans. To answer this profoundly important question, we advocate for the scientific community to perform the necessary research in this arena.

## 9. Conclusion

The *DRD2* gene has been extensively investigated in various neuropsychiatric disorders. Numerous international studies have been performed since the initial association of the *DRD2 Taq A1* allele with severe alcoholism in 1990. In our opinion, the primary cause of negative reports regarding the association of various *DRD2* gene polymorphisms is the inadequate screening of controls, failing to eliminate many hidden RDS behaviors. Moreover, pleiotropic effects of *DRD2* variants have been observed in neurophysiological, neuropsychological, stress response, social stress defeat, MD, and gambling disorder contexts, where epigenetic DNA methylation and negative histone post-translational methylation have been identified, as discussed in this commentary. As of October 19, 2022, there are 70 articles focusing on DNA methylation and 20 articles on histone methylation are listed in PUBMED. Importantly, Blum and Noble characterized the *DRD2 Taq A1* allele not as specific to alcoholism but as a generalized reward gene. Therefore, it now behooves the field to find ways to either use effector moieties to edit the neuroepigenetic insults or possibly harness the idea of potentially removing negative mRNA-reduced expression by inducing dopamine homeostasis. This represents a futuristic laudable goal.

## Figures and Tables

**Table 1.** Epigenetics of substance use disorder

Type of substance	Findings	References
Cannabis	Higher histone methylation onto *DRD2* gene with family history	Oyaci *et al*.^105^
	DNA methylation on *DRD2* promotor	Gerra *et al*.^106^
	Prenatal Δ-9- tetrahydrocannabinol (THC) exposure with an increase in histone methylation *DRD2* gene	DiNieri *et al*.^107^
Tobacco	DNA methylation on *NCAM1-TTC12-ANKK1-DRD2* gene cluster associated with risk for cigarettes per day	Liu *et al*.^109^
Alcohol	Enhanced global histone methylation	Pandey *et al*.^111^
	*DRD2* promoter DNA methylation was positively associated with responses to alcohol severity	Bidwell *et al*.^112^
	*DRD2* DNA methylation was significantly associated with alcohol problem severity	Hagerty *et al*.^114^
	DNA methylation at the *DRD2* gene was significantly increased in association with familial high-risk status	Hill and Sharma^116^
	Craving was significantly associated with *DRD2*-gene methylation	Hillemacher *et al*.^117^
Psychostimulants	METH-treated mice also showed (i) decreased global levels of total H3ac and H4ac and increased global levels of 5-methylcytosine (5-mC) and (ii) decreased H3ac enrichment at promoters of *DRD2*	González *et al*.^121^
	In both human and animal self-administration experiments, the histone mark protein-R- methyltransferase-6 (PRMT6) and asymmetric demethylation of R2 on histone H3 (H3R2me2a) decreased in the rodent and cocaine-dependent human NAc.	Damez-Werno *et al*.^122^
	METH induced a decrease in DNA methylation of a number of dopamine-related genes (i.e., *DRD3, DRD4*, and *COMT)* in patients with METH psychosis but not in non-METH psychosis patients.	Nohesara *et al*.^124^
Opioids	Specifically, rs4867798-CpG_174872884 and rs5326-CpG_174872884 in the *DRD1* gene showed hypermethylation	Zhang *et al*.^125^
